# *Veronicastrum axillare* Alleviates Lipopolysaccharide-Induced Acute Lung Injury via Suppression of Proinflammatory Mediators and Downregulation of the NF-*κ*B Signaling Pathway

**DOI:** 10.1155/2016/7934049

**Published:** 2016-11-06

**Authors:** Quanxin Ma, Kai Wang, Qinqin Yang, Shun Ping, Weichun Zhao, Qiyang Shou, Weimin Zhou, Minli Chen

**Affiliations:** ^1^Animal Experimental Research Center, Zhejiang Chinese Medical University, Hangzhou 310053, China; ^2^College of Animal Sciences, Zhejiang University, Hangzhou 310058, China; ^3^Institute of Apicultural Research, Chinese Academy of Agricultural Sciences, Beijing 100093, China; ^4^Zhejiang Institute of Traditional Chinese Medicine, Hangzhou 310007, China; ^5^College of Bioengineering, Zhejiang Chinese Medical University, Hangzhou 310053, China

## Abstract

*Veronicastrum axillare* is a traditional medical plant in China which is widely used in folk medicine due to its versatile biological activities, especially for its anti-inflammatory effects. However, the detailed mechanism underlying this action is not clear. Here, we studied the protective effects of* V. axillare* against acute lung injury (ALI), and we further explored the pharmacological mechanisms of this action. We found that pretreatment with* V. axillare* suppressed the release of proinflammatory cytokines in the serum of ALI mice. Histological analysis of lung tissue demonstrated that* V. axillare* inhibited LPS-induced lung injury, improved lung morphology, and reduced the activation of nuclear factor-*κ*B (NF-*κ*B) in the lungs. Furthermore, the anti-inflammatory actions of* V. axillare* were investigated* in vitro*. We observed that* V. axillare* suppressed the mRNA expression of interleukin-1*β* (IL-1*β*), IL-6, monocyte chemotactic protein-1 (MCP-1), cyclooxygenase-2 (COX-2), and tumor necrosis factor-*α* (TNF-*α*) in RAW264.7 cells challenged with LPS. Furthermore, pretreatment of* V. axillare in vitro* reduced the phosphorylation of p65 and I*κ*B-*α* which is activated by LPS. In conclusion, our data firstly demonstrated that the anti-inflammatory effects of* V. axillare* against ALI were achieved through downregulation of the NF-*κ*B signaling pathway, thereby reducing the production of inflammatory mediators.

## 1. Introduction

Acute lung injury (ALI) is a prototypical inflammatory disease. ALI's most significant pathological feature is the acute lung inflammation, including inflammatory cell recruitment and the release of proinflammatory mediators [1, 2]. Lipopolysaccharide (LPS) is the major component of the cell wall in Gram-negative bacteria. LPS can be used to produce a classical animal model of ALI associated with the activation of monocytes, overflow of pulmonary neutrophils, increased levels of alveolar-capillary permeability, and diffuse alveolar damage [3]. During the initiation of the host defense process, LPS is recognized by Toll-like receptor 4 (TLR4), which then activates several intracellular signaling pathways, among which nuclear factor-*κ*B (NF-*κ*B) pathway is the predominant one. The activation of NF-*κ*B eventually leads to the upregulation of several inflammatory factors [4]. Therefore, interfering with NF-*κ*B signaling pathway is a promising therapeutic regimen for the treatment of acute inflammatory disease, like ALI.

*Veronicastrum axillare* is a folk medicine which is widely used in many regions in East Asia to treat ascites, burns, and snakebites, among other ailments [5]. Our previous work indicated that* V. axillare* significantly inhibits ethanol-induced gastric ulcers [6]. Recently, it was shown that the ethyl acetate (EtOAc) extracts of* V. axillare* displayed potent anti-inflammatory activity* in vivo* using xylene-induced mouse ear edema model [7]. Nevertheless, effects of* V. axillare* against ALI and potential anti-inflammatory mechanisms are poorly understood. Here, we assessed the anti-inflammatory effects of* V. axillare in vivo* employing LPS-induced ALI mice. We also explored the underlying mechanisms of this action, including the possible inhibition on the activation of macrophages.

## 2. Materials and Methods

### 2.1. Animals and Chemical Reagents

Male ICR mice (20 ± 2 g) were purchased from the Shanghai Laboratory Animal Research Center and housed in the Animal Experimental Center, Zhejiang Chinese Medical University. Four mice in one cage were housed in a room with controlled lighting (12 h light/dark cycle) and temperature (20 ± 2°C), and the air was filtered by a ventilation system with relative humidity of 50%. Mice were fed with a standard laboratory diet (AIN-93 formulation for experimental animals, Xietong Biotechnology, Nanjing, China) and water* ad libitum* and received careful care to fulfill the requirement of animal welfare during experimental periods. The animal experimental protocols described in this study were in accordance with the regulations from the Animal Experimental Center, Zhejiang Chinese Medical University, and were approved by Zhejiang Chinese Medicine University Animal Care Committee.

LPS (*Escherichia coli* O111:B4) and alkaline phosphatase-conjugated secondary antibody (anti-rabbit IgG) were purchased from Sigma (St. Louis, MO, USA). Primary rabbit monoclonal antibodies against phospho-I*κ*B-*α* (pS36), I*κ*B-*α*, and *β*-tubulin were purchased from Epitomics (Burlingame, CA, USA). Primary rabbit monoclonal antibodies phospho-NF-*κ*B p65 (Ser536) were purchased from Cell Signaling (Beverly, MA, USA). All the other reagents were obtained from Sangon Biotechnology (Shanghai, China) unless indicated specifically in each section.

### 2.2. Preparation of* V. axillare* Aqueous Extract

Whole* V. axillare* plants were gathered in Lishui, Zhejiang Province, China, and identified by Professor Zhensheng Yao (School of Pharmacy, Zhejiang Chinese Medical University, China). The aqueous extract of* V. axillare* was prepared from air-dried and powdered whole plants. In brief, the powdered plants were soaked for 0.5 h in 20°C with 1 L water. The samples were then boiled for 1.5 h with gentle heat three times in 100°C. The decoction was then concentrated to 140 g/L for storage at 4°C in the fridge in the dark. The extract was diluted to the corresponding concentration with pure water before use [6].

### 2.3. Experimental Protocols for the Animal Studies

After 4 d of adaptation, the mice were randomly divided into five groups (*n* = 8) as follows: (1) control group (normal saline, 10 mL/kg orally); (2) LPS-induced model group (LPS, 1 mg/kg via tail vein injection); (3)* V. axillare* low-dose group (*V. axillare*, 300 mg/kg orally and LPS injection); (4)* V. axillare* high-dose group (*V. axillare*, 1200 mg/kg orally and LPS injection); and (5) dexamethasone-treated group (DEX and LPS injection). The mice were orally pretreated with* V. axillare* (300 or 1200 mg/kg) for 3 consecutive days, while mice from other groups (control, LPS, and DEX group) received the normal saline solutions. The mice in the DEX-treated group were given DEX orally (2 mg/kg) 1 h before LPS challenge. One hour after the final* V. axillare* treatment, all animals except for the control group were challenged with LPS via tail vein to induce ALI. The mice were sacrificed 6 h after the LPS tail injection [8]. Blood samples were collected and centrifuged at 3000 rpm for 10 min at 4°C to obtain serum, which was then stored at −80°C for further experiments. Lung tissues were harvested simultaneously from mice and soaked in 10% formalin.

### 2.4. Histological Evaluation of the Lungs

Mice lung tissues were immediately dehydrated and embedded in paraffin. Paraffin sections (4 *μ*m, 6–10 per lung) were sliced and stained with hematoxylin and eosin (H&E) using a standard conventional method. Pathological sections were observed under a light microscope at 400x amplification. Photos were taken by the attached camera (Nikon).

Immunostaining was performed according to Lin's experimental method [9]. The sections were blocked with BSA (Sangon, Shanghai) and then incubated with NF-*κ*B primary antibody (Santa Cruz) overnight at 4°C. Horseradish peroxidase- (HRP-) conjugated secondary antibodies were used for 1 h incubation. Finally, the sections were treated with* Dolichos biflorus* agglutinin (DBA) as a color reagent and counterstained with hematoxylin. The sections were viewed with the light microscope, and the pathological pictures were analyzed by ImageJ software.

### 2.5. Cell Culture and Cell Viability Assay

The murine macrophage RAW264.7 cell line was donated by Professor Fuliang Hu (College of Animal Sciences, Zhejiang University, China). The cells were cultured in DMEM supplemented with 10% fetal bovine serum (FBS), 100 U/mL penicillin, and 100 g/mL streptomycin at 37°C in 5% CO_2_ atmosphere. The viability of RAW264.7 cells incubated with* V. axillare* was determined by using cell counting kit-8 (CCK-8, Dojindo, Japan). RAW264.7 cells were seeded in 96-well plates at a density of 10^4^ cells/well. After incubating for 24 h, the cells were treated with various concentrations of* V. axillare* extract for an additional 24 h. CCK-8 reagent (10 *μ*L) was added to the cells and then further incubated for 2 h. The OD values of each well were measured using a microplate reader at 450 nm (Bio-Rad Model 550, CA).

### 2.6. Measurement of Inflammatory Cytokines

Levels of various inflammatory cytokines, including interleukin-6 (IL-6), IL-10, monocyte chemotactic protein-1 (MCP-1), interferon-*γ* (IFN-*γ*), tumor necrosis factor-*α* (TNF-*α*), and IL-12p70, were assessed using a cytometric bead array (CBA) mouse inflammation kit (BD Biosciences). Serum samples were diluted 25-fold as instructed by the manufacturer.

### 2.7. RNA Extraction and Quantitative Real-Time PCR (qPCR)

Total cellular RNA was extracted using an RNA extraction kit (Aidlab Biotechnologies, Beijing, China) following the directions of the manufacturer. For each sample, cDNA was synthesized using the Prime Script RT reagent kit (TaKaRa, Dalian, China). The primer sequences are shown in Table 1 and were synthesized by Sangon Biotechnology Co., Ltd. qPCR was executed in an automated thermal cycler (Eppendorf, Hamburg, Germany) in a final volume of 25 *μ*L (2 *μ*L cDNA, 12.5 *μ*L SYBR® Premix Ex Taq, 1 *μ*L of each primer, 10 *μ*mol/L, and 8.5 *μ*L ddH_2_O). The cycling reaction was performed using a standard two-step PCR reaction.

### 2.8. Western Blotting Analysis

For western blotting analysis, cells were washed twice with chilled PBS and then lysed in lysis buffer (50 mM Tris, pH 7.5, 150 mM NaCl, 0.5% NP-40, 10% glycerol, 2 mM DTT, 1 mM leupeptin, and 1 mM PMSF) on ice for 30 min. Obtained cell lysates were cleared by centrifugation at 12,000 ×g for 15 min at 4°C. Cellular proteins (30 *μ*g) were then mixed with sampling buffer and further boiled for 5 min at 95°C. Then, proteins (10 *μ*g) were separated by SDS-PAGE gels and then transferred to polyvinylidene fluoride (PVDF) membranes. The membranes were blocked with 5% nonfat milk at room temperature and then probed with primary polyclonal antibodies overnight at 4°C. After that, the membranes were washed with TBST and then incubated with secondary antibody for 1 h at room temperature. Then, the membranes were washed again three times in TBST. Finally, the immune reactive protein bands on the membrane were visualized using 10 mL alkaline phosphatase in a color development buffer.

### 2.9. Statistical Analysis

All values were presented as the mean ± SD for at least three independently performed experiments. The data's statistical comparison was performed by one-way analysis of variance (ANOVA), followed by the *t*-test. Statistical significance was set at *p* < 0.05. All statistical tests were performed using SPSS 19.0.

## 3. Results

### 3.1. *V. axillare* Reduced LPS-Induced Augmentation of Serum Cytokines and Attenuated the Pathological Injuries in Mouse Lungs

In order to assess the protective effect of* V. axillare* in mice with ALI, serum inflammatory cytokines and pulmonary histopathology were evaluated.* V. axillare* (300 or 1200 mg/mL) was administered intragastrically 3 days before LPS injection. As demonstrated in Table 2, LPS stimulation increased inflammatory cytokine production, including IL-6, MCP-1, TNF-*α*, and IL-12p70 in mouse serum. However, the following pretreatment with* V. axillare* (1200 mg/kg) and DEX (2 mg/kg) dramatically inhibited the release of inflammatory cytokines in ALI mice.

**Figure 1:**
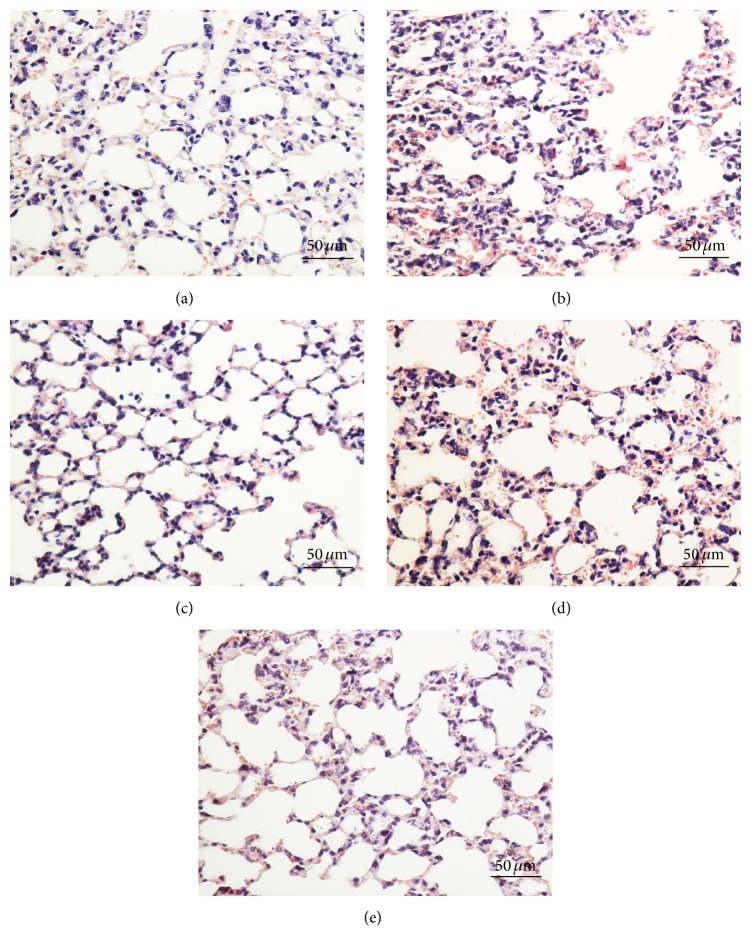
Effects of* V. axillare* on histopathological changes in lung tissues in LPS-induced ALI mice. Representative lung sections from (a) control group; (b) LPS-induced ALI model group; (c) mice injected with LPS and treated with DEX (2 mg/kg); (d and e) mice injected with LPS and given* V. axillare* (400 or 1200 mg/kg) are shown. Histological changes were evaluated by H&E staining and microscopy (original magnification: 200x, scale bar: 50 *μ*m).

After staining with H&E, histopathological changes in the lungs of each group were observed. A lot of apparent structural damage was found in the pulmonary tissue from the mice of LPS-treated model group, including increased alveolar wall thickness, inflammatory cell aggregation, and pulmonary hemorrhage (Figure 1). These injuries were not apparent in the control group. Pretreatment with 1200 mg/kg* V. axillare* and 2 mg/kg DEX significantly inhibited LPS-induced histological changes. These results indicated that* V. axillare* protected against LPS-induced lung tissue damage.

**Figure 2:**
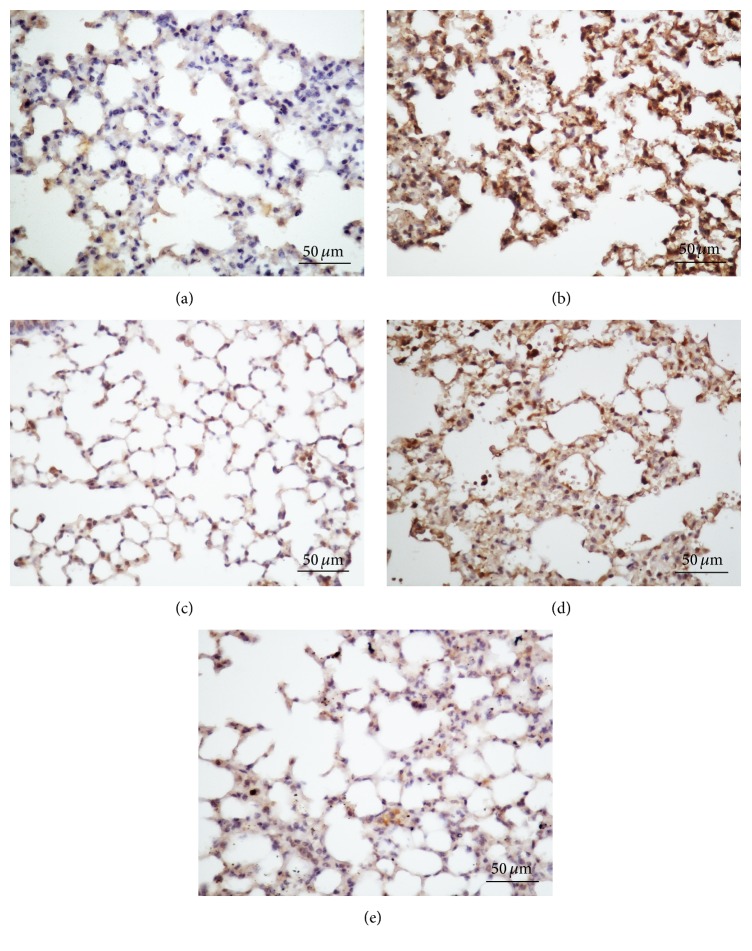
Representative immunohistochemical analysis of NF-*κ*B from (a) the control group, (b) the LPS-treated model group, (c) mice treated with DEX (2 mg/kg), and (d and e) mice treated with* V. axillare* (400 or 1200 mg/kg). Magnification: 200x, scale bar: 50 *μ*m.

We further evaluated NF-*κ*B activation in the lung sections using immunohistochemistry. The NF-*κ*B p65 subunit was stained brown and was primarily localized in the nuclei of inflammatory cells (Figure 2). The expression of NF-*κ*B in the LPS-treated model group was higher than in the control group. Both* V. axillare* and DEX treatment reduced the positive percentage and density of the NF-*κ*B p65 subunit significantly (*p* < 0.05), indicating that both* V. axillare* and DEX may have inhibited the activation of NF-*κ*B in the lungs.

### 3.2. Effects of* V. axillare* on RAW264.7 Cell Viability

**Figure 3:**
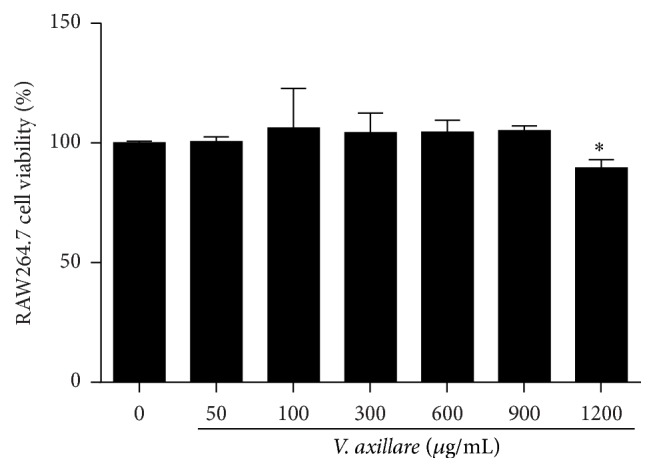
Effects of* V. axillare* on cell viability in RAW264.7 cells. Various concentrations of* V. axillare* were treated to the cells for 24 h, and the cell viability was measured by CCK-8 assay, as described in the Materials and Methods. The values represent the mean ± SD from three independent experiments (^*∗*^ *p* < 0.05 indicates a significant difference versus untreated control cells by Student's *t*-test).

In order to observe whether* V. axillare* had any toxic effects on cells and to find a suitable concentration for application in the subsequent* in vitro* experiments, we examined the effects of* V. axillare* on cell viability in RAW264.7 cells using a CCK-8 assay.  Figure 3 shows that there were no cytotoxic effects in RAW264.7 cells treated with* V. axillare* within proper concentrations (up to 900 *μ*g/mL). Based on these results, we selected suitable concentration ranges during the following* in vitro* experiments.

### 3.3. *V. axillare* Modulated Several Key Inflammatory-Related Gene Expressions in LPS-Stimulated RAW264.7 Macrophages

**Figure 4:**
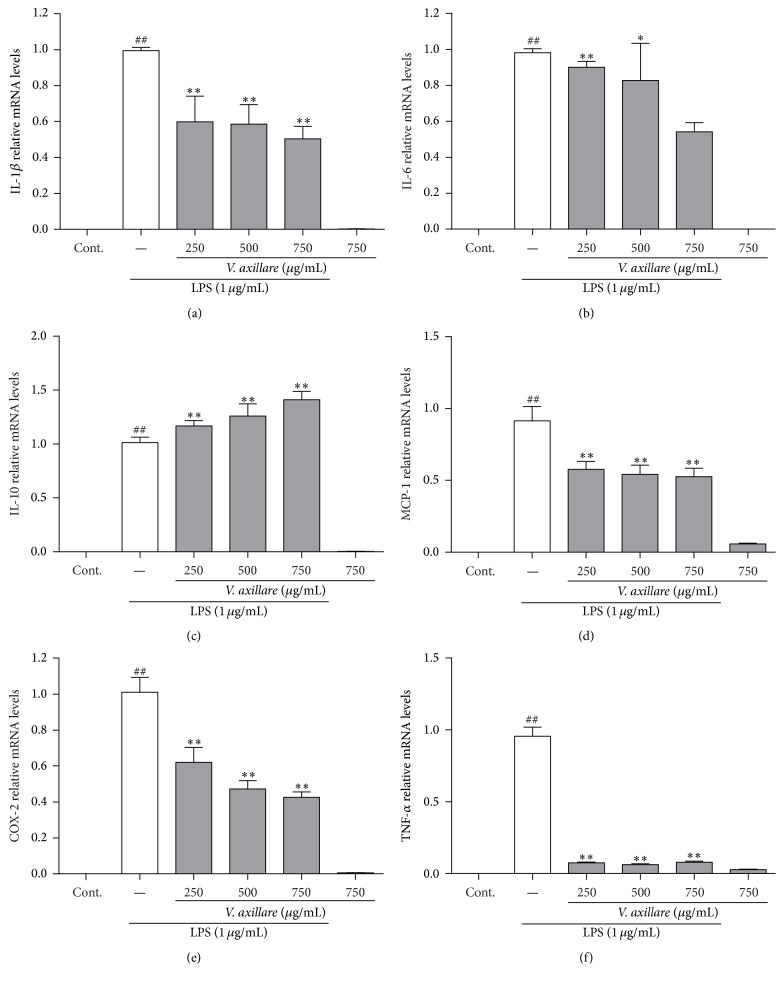
Effects of* V. axillare* on the gene expression of key proinflammatory genes in LPS-stimulated RAW264.7 cells. Various concentrations of* V. axillare* (250, 500, and 750 *μ*g/mL) were treated to the cells for 1 h before incubation with LPS (1 *μ*g/mL) for 6 h. Additionally, one group was treated with* V. axillare* (750 *μ*g/mL) alone to observe the effects of* V. axillare* on the RAW264.7 without LPS stimulation. The mRNA expression of (a) IL-1*β*, (b) IL-6, (c) IL-10, (d) MCP-1, (e) COX-2, and (f) TNF-*α* was analyzed by qRT-PCR. Data are presented as the mean ± SD from three independent experiments. Student's *t*-test was performed to compare individual groups (^*∗*^ *p* < 0.05 and ^*∗∗*^ *p* < 0.01 indicate a significant difference compared with the LPS group; ^##^ *p* < 0.01 indicates a significant difference compared with the untreated group).

To assess the effect of* V. axillare* on inflammatory mRNA expression in LPS-stimulated RAW264.7 cells, we analyzed the levels of several inflammatory genes (IL-1*β*, IL-6, TNF-*α*, MCP-1, and COX-2) through real-time PCR. The RAW264.7 cells were stimulated with LPS alone or LPS and* V. axillare* for 6 h. We selected this time point because the mRNA expression of the proinflammatory genes reached a peak at this point according to our previous studies [10]. As shown in Figure 4, after stimulation with LPS, the expression of IL-1*β*, IL-6, TNF-*α*, MCP-1, and COX-2 was upregulated. Importantly, this upregulation was not observed in RAW264.7 cells cultured with* V. axillare* alone. Pretreatment with* V. axillare* (250, 500, and 750 *μ*g/mL) significantly reduced the mRNA expression of these genes in a dose-dependent manner.

### 3.4. *V. axillare* Inhibits LPS-Induced Phosphorylation of p65 and Prevented I*κ*B-*α* Degradation in RAW264.7 Macrophages

**Figure 5:**
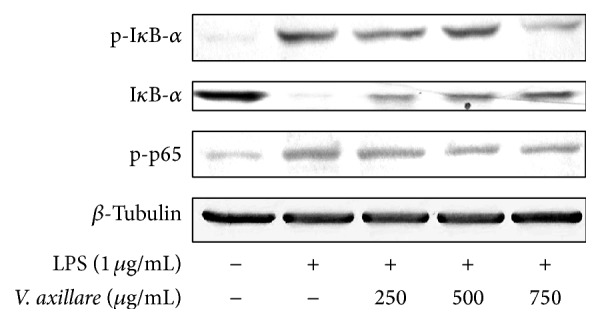
*V. axillare* inhibits LPS-induced phosphorylation of p65 and prevented I*κ*B-*α* degradation in RAW264.7 macrophages. Cells were pretreated with* V. axillare* for 1 h before incubation with LPS for 30 min. Levels of phosphorylated p65 and total and phosphorylated I*κ*B-*α* were determined by western blot analysis. *β*-Tubulin was used as a loading control.

The genes repressed by* V. axillare* are normally controlled by the transcription factor NF-*κ*B. Phosphorylation and degradation of I*κ*B-*α* lead to the phosphorylation of NF-*κ*B p65 subunit. This phenomenon enables the release and translocation of the free NF-*κ*B into the nucleus, where it binds specifically to the *κ*B binding sites on DNA [11]. Therefore, we examined whether* V. axillare* altered these processes. As shown in Figure 5, LPS stimulation decreased the I*κ*B-*α* protein level in untreated cells, which was consistent with the increased level of phosphorylated p65 and I*κ*B-*α*. In contrast, pretreatment with* V. axillare* at 250, 500, and 750 *μ*g/mL significantly reduced the phosphorylation of p65 and I*κ*B-*α* induced by LPS, indicating that* V. axillare* does exert an anti-inflammatory effect through the inhibition of LPS-induced NF-*κ*B signaling pathway.

## 4. Discussion

In this study, we illustrated that, after pretreatment with* V. axillare*, the expression of proinflammatory cytokines (MCP-1, IFN-*γ*, TNF-*α*, IL-6, and IL-12p70) in the serum of mice with LPS-induced ALI was notably reduced. We also found that* V. axillare* improved pulmonary histology in these mice. These findings collectively suggest that* V. axillare* may be effective for ALI prevention induced by LPS. In order to research the mechanisms of* V. axillare* action, we used RAW264.7 cells to evaluate changes in inflammatory mediators, cytokines, and signaling pathways.

During the progression of inflammation, proinflammatory cytokines such as TNF-*α* and IL-6 are released by macrophages to protect the body from tissue injury or infection [12]. Furthermore, COX-2 is known as the rate-limiting enzyme that not only participates in the synthesis of prostaglandin E2, but also contributes to the inflammatory reaction [13]. The activation of macrophages leads to high expression level of MCP-1. The release of MCP-1 will cause the migration of tissue macrophages and blood monocytes to the site of inflammation, thereby promoting chronic inflammatory dysfunctions. Therefore, the mRNA expression levels of these inflammatory mediators are significant indicators during inflammation. In our study, the stimulation of LPS to the RAW264.7 cells led to considerable changes of several proinflammatory genes, including IL-1*β*, IL-6, COX-2, MCP-1, and TNF-*α*, which correlate well with previous studies [14].* V. axillare* also decreased the mRNA expression of these genes. These results provide direct evidence that* V. axillare* inhibited the development of inflammation through decreasing proinflammatory gene expressions in LPS-stimulated macrophages.

LPS is a TLR4 agonist in macrophages [15]. The association between TLR4 and LPS results in the fast activation of NF-*κ*B [16]. The expression of various cytokines, chemokines, cell-adhesion molecules, and growth factors in the lungs is closely regulated by the activation of NF-*κ*B [17]. Thus, the suppression of the proinflammatory mediators by the modulation of intracellular signaling in macrophages could be used for treating LPS-induced pulmonary diseases [18]. Our* in vitro* data suggest that NF-*κ*B was activated after LPS stimulation, evidenced by the degradation of I*κ*B-*α* and the phosphorylation of NF-*κ*B p65, which correlate well with previous findings [6, 14]. These responses were prevented by* V. axillare* pretreatment. Similar results were acquired by immunohistochemical staining of less activated NF-*κ*B* in vivo*. These results suggest that the anti-inflammatory effect of* V. axillare* might be mediated via the inhibition of the NF-*κ*B signaling pathways.

Macrophages play an important role during the innate immunity of the host response against many types of infections by producing various inflammatory mediators and adhesion molecules, which activate the immune system [19]. A large number of infection-associated disorders have been linked to the aberrant activation of macrophages, including ALI, sepsis, and septic shock. Therefore, agents which can inhibit the production/release of those inflammatory mediators can be developed as potential anti-inflammatory agents [20]. To further understand the underlying mechanisms of the anti-inflammatory effects of* V. axillare*, we evaluated the effects of* V. axillare *on the LPS-induced TLR4 signaling pathway in RAW264.7 cells. Previous mechanistic studies indicate that LPS-stimulated TLR4 activation was the most prominent mechanism of macrophage activation and can activate two different signaling pathways: the MyD88-dependent and the TRIF-dependent pathways [21, 22]. The MyD88-dependent pathway plays a critical role in the regulation of macrophage activation, thereby promoting the activation of MAPKs and NF-*κ*B. It is known that LPS-induced activation of MAPKs and NF-*κ*B leads to the expression of several proinflammatory mediators in macrophages [23, 24]. We demonstrated that* V. axillare* extracts significantly suppressed LPS-induced phosphorylation of p65 and I*κ*B-*α* in RAW264.7 macrophages, which was consistent with our* in vivo* data.

In terms of its chemical composition, the previous studies have found that* V. axillare* contains flavonoids, phenols, phenylpropanoids, terpenoids, tannins, and so forth [5]. Recently, three new phenylpropanoid glycosides isolated from* V. axillare* exerted strong anti-inflammatory effects, including procumboside A. It is the major anti-inflammatory constituent of the EtOAc fraction of* V. axillare*. Interestingly, the anti-inflammatory effect of procumboside A seems to be stronger than that of dexamethasone which is also used as a positive drug in a previous study [7]. Nevertheless, the interactions among active compounds in the natural products are complex with potential synergistic/antagonistic effects, which attracts great interest in TCM research. Hence, it is valuable to study the interaction among the aforementioned compounds in* V. axillare* in the future.

Taken together, our study confirmed that the aqueous extract of* V. axillare* exerted strong anti-inflammatory effects by attenuating LPS-induced lung injury.* V. axillare* prevented the overproduction of inflammatory cytokines in ALI mice. Histological analysis indicated that* V. axillare* significantly improved lung injury and decreased pulmonic NF-*κ*B activation. We also noticed that* V. axillare* could downregulate the mRNA expressions of IL-1*β*, IL-6, IL-10, MCP-1, COX-2, and TNF-*α* in LPS-activated RAW264.7 cells. These effects seem to be mediated, at least in part, by the inhibition of NF-*κ*B activation, which provides first-hand evidence for the development of anti-inflammatory drugs in the future. Further studies about the active components in* V. axillare* are required for better understanding the protective effects against ALI.

## Figures and Tables

**Table 1:** Primers used for qRT-PCR experiments.

Gene	Primer sequence	GenBank accession number
IL-1*β*	5′-CCAACAAGTGATATTCTCCATGAG-3′	NM_008361.3
	5′-ACTCTGCAGACTCAAACTCCA-3′	
IL-6	5′-CTCTGCAAGAGACTTCCATCC-3′	NM_031168.1
	5′-GAATTGCCATTGCACAACTC-3′	
IL-10	5′-CTATGCTGCCTGCTCTTACTG-3′	NM_010548.2
	5′-CAACCCAAGTAACCCTTAAAGTC-3′	
TNF-*α*	5′-CCACGCTCTTCTGTCTACTG-3′	NM_013693.2
	5′-ACTTGGTGGTTTGCTACGAC-3′	
MCP-1	5′-AAGAAGCTGTAGTTTTTGTCACCA-3′	NM_011333.3
	5′-TGAAGACCTTAGGGCAGATGC-3′	
COX-2	5′-GAAATATCAGGTCATTGGTGGAG-3′	NM_011198.3
	5′-GTTTGGAATAGTTGCTCATCAC-3′	
GAPDH	5′-GAGAAACCTGCCAAGTATGATGAC-3′	NM_008084.2
	5′-TAGCCGTATTCATTGTCATACCAG-3′	

**Table 2:** Effect of *V. axillare* on serum inflammatory cytokines in LPS-challenged mice^a^.

Group	Dose (mg/kg)	MCP-1 (ng/mL)	IFN-*γ* (ng/mL)	TNF-*α* (pg/mL)	IL-6 (ng/mL)	IL-10 (pg/mL)	IL-12p70 (pg/mL)
Control	—	ND	ND	ND	ND	ND	ND
Model	—	98.27 ± 10.69	5.22 ± 0.31	1897.57 ± 93.31	96.00 ± 10.66	237.15 ± 2.04	100.60 ± 8.08
*V. axillare*	300	81.31 ± 6.52^*∗*^	6.44 ± 0.62	1290.10 ± 250.03^*∗*^	83.17 ± 8.17	225.95 ± 9.78	63.72 ± 2.57^*∗*^
*V. axillare*	1200	60.45 ± 2.42^*∗∗*^	3.02 ± 0.09^*∗∗*^	711.36 ± 32.64^*∗∗*^	33.82 ± 4.47^*∗∗*^	118.84 ± 7.28^*∗∗*^	25.15 ± 2.04^*∗*^
Dexamethasone	2	67.93 ± 1.18^*∗∗*^	2.69 ± 0.64^*∗∗*^	850.81 ± 58.44^*∗∗*^	52.05 ± 7.37^*∗∗*^	180.21 ± 11.53^*∗∗*^	26.96 ± 0.52^*∗∗*^

^a^Values are the means ± SD (*n* = 8 for each group). ^*∗*^ *p* < 0.05 and ^*∗∗*^ *p* < 0.01 indicate a significant difference compared to the model group. ND: not detected.
